# The impact of maternal gum disease on pregnancy outcomes using PRAMS data

**DOI:** 10.1371/journal.pone.0325588

**Published:** 2025-07-07

**Authors:** Khadijat Kofoworola Adeleye, Joohyun Chung

**Affiliations:** Elaine Marieb College of Nursing University of Massachusetts Amherst, Amherst, United States of America; International Medical University, MALAYSIA

## Abstract

**Background:**

Gum disease during pregnancy is not just a dental issue but a significant public health concern with potential implications for maternal and child health. This study aims to estimate the national prevalence of gum disease among pregnant women and examine the association of maternal gum disease during pregnancy and pregnancy outcomes.

**Methods:**

A retrospective case-control study used the PRAMS (phase 8, 2016–2020) dataset. Propensity score matching was employed to match cases in a 1:3 ratio. Logistic regression was used to test the associations between maternal gum disease, low birth weight, and small gestational age.

**Results:**

The study revealed a prevalence rate of 207 cases per 100,000 pregnant women. Notable differences were observed, with higher prevalence rates among younger women (20–24 yrs), racial minorities (Black women), and those with lower educational attainment (9–12 grade, no diploma). Maternal gum disease was associated with an increased risk of small for gestational age (SGA) and falls below the 10th percentile (OR = 2.43, 95% CI: 1.56–3.75, p < 0.001), having babies with birthweight below 2500g are 3.51 times higher (OR = 3.51, 95% CI: 2.39–5.16, p < 0.001) compared to those who do not have gum disease.

**Conclusion:**

The complex relationship between maternal oral health and adverse pregnancy outcomes necessitates immediate and comprehensive research to understand and address this issue, highlighting the immediate need for action to prevent potential health risks.

## Introduction

Maternal oral health is a critical component of overall maternal well-being, with potential implications for the mother and the developing fetus^.^ One prevalent oral health condition that has garnered attention in the context of pregnancy is periodontal disease [1]. According to the Centers for Disease Control, 60–70% of pregnant women have some form of gum disease [2]. In Spain, a higher number of women had periodontal and gingival disease in the third trimester of gestation [3]^.^ In a study conducted in Malaysia, about 56.3% of pregnant women complained of oral gingivitis during pregnancy [4]. Similarly, in India and Thailand, the majority of pregnant women had poor oral health and gingivitis [5,6]. Understanding the prevalence and impact of oral health issues among pregnant women and their potential impact on pregnancy outcomes is crucial in addressing the broader public health implications of these conditions.

Pregnancy introduces physiological changes that can affect oral health, including hormonal fluctuations that increase the risk of periodontal disease and gingivitis [7–10]. Research suggests these changes may increase susceptibility to oral health issues such periodontal disease [11,12]. Despite the known risks, oral health during pregnancy is often overlooked, leading to a high prevalence of oral health issues among pregnant women.

Recent studies have presented divergent figures regarding dental visit rates during pregnancy in different regions. Notably, in the United States, particularly in California and Utah, there has been an upward trend in dental visits during pregnancy [13,14]. Previous studies reported lower racial disparities among birthing individuals who receives dental cleaning during pregnancy [15,16]. Additionally, research conducted in Upstate New York highlighted unmet oral health needs among underserved pregnant women in the region [17]. These diverse findings underscore the importance of addressing disparities in access to dental care during pregnancy and highlight the significance of promoting oral health among pregnant women.

Several factors have been identified as influential in shaping oral healthcare behaviors during pregnancy. The perception that dental treatment during pregnancy is unsafe was essential in limiting dental care utilization among pregnant women [18–20]. Additionally, barriers such as financial constraints, lack of access to food programs, social assistance, access to care difficulties, and limited oral health literacy were identified as significant challenges faced by a low socioeconomic status (SES) in the United States [21]. Additionally, women who have experienced prenatal physical intimate partner violence (IPV) and multiple forms of disabilities around the time of pregnancy are at elevated risk of not having dental insurance and not undergoing dental prophylaxis during pregnancy, highlighting the complex interplay between social, economic, and health factors in shaping access to oral healthcare for pregnant women [22–24].

While there is a growing body of research exploring the link between maternal gum disease and adverse pregnancy outcomes, there is a notable gap in understanding how social determinants of health influence the occurrence and progression of gum disease during pregnancy. The existing literature predominantly focuses on establishing associations between gum disease and adverse pregnancy outcomes, often overlooking the intricate web of social factors that may serve as potential determinants of maternal oral health [6,23,25,26]. According to the Centers for Disease Control and Prevention (CDC), poor oral health during pregnancy can lead to poor health outcomes for the mother, including heart disease and diabetes, and the baby, including preterm birth and low birth weight [27,28]. Studies have indicated that the presence of periodontal disease and inadequate dental care during pregnancy may contribute to maternal stress and immune dysregulation, exacerbating existing systemic conditions and increasing the risk of preterm birth [29–34]. Understanding these determinants is crucial for developing targeted interventions and preventive measures that address the oral health aspect and the broader psychosocial context in which pregnant women navigate their healthcare.

This study examined the relationship between maternal gum disease and pregnancy outcomes using PRAMS data. This data is essential to filling existing gaps in evidence, informing targeted interventions, and enhancing prenatal care practices to safeguard the health of mothers and baby during this critical period.

**Aim:** To assess the impact of maternal gum disease on pregnancy outcomes (low birth weight and small gestation under the 10th percentile)

### Research questions

1. What is the prevalence of gum disease among pregnant women in the USA?
2. What is the association between socioeconomic status (e.g., education, income, age) and gum disease among pregnant women in the United States?
3. What are the relationships between gum disease and pregnancy outcomes (low birth weight and small gestation under the 10th percentile) among pregnant women?

## Methods

### Study design and data sources

In this case-control study, we utilised the Pregnancy Risk Assessment Monitoring System (PRAMS) dataset to examine the relationship between gum disease and adverse birth outcomes among pregnant women.

### Data sources

The Pregnancy Risk Assessment Monitoring System (PRAMS phase 8 (2016–2021) is a joint research project between state, territorial, or local health departments and the Centers for Disease Control and Prevention (CDC), Division of Reproductive Health. PRAMS collects state-specific, population-based data through surveys that address various aspects of maternal health, including prenatal care, behaviors, and experiences. The system plays a crucial role in identifying groups of women and infants at high risk for health problems, monitoring changes in health status, and measuring progress toward improving the health of mothers and infants. By providing site-specific data, PRAMS enables comparisons among participating sites using standardized data collection methods.

### Case-control selection

We have utilised the Pregnancy Risk Assessment Monitoring System (PRAMS) dataset, phase 8 (2016–2021), which comprises data collected from 78166 pregnant women. The sample was allocated into two groups, according to self-reported gum disease during pregnancy as follows;

**Case:** 162 women were identified as having gum disease during pregnancy, based on their responses to the question, “Health problem during pregnancy – Gum disease.” They were meticulously selected using a propensity score-matched analysis to ensure a robust comparison. This method allowed us to match each case of gum disease with control subjects who did not have gum disease but shared similar characteristics, thereby minimizing confounding variables. The matching was based on several key variables, including age, race, education, marital status, smoking habits, prenatal care index, and method of payment for healthcare services.

To enhance the precision of our findings, a matching ratio of 1:3 (gum disease patients to control subjects) was employed. This ratio was chosen to ensure a sufficient sample size for the control group, increasing the study’s statistical power. As a result, 486 control subjects were selected, ensuring that the baseline characteristics between the gum disease and control groups were balanced.

**Covariates:** Seven covariates variables were examined according to the study’s objective and based on a comprehensive literature review and their availability in the PRAMS datasets: maternal age at delivery (≤17, 18–24, 25–29, 30–34, ≥ 35 years), maternal race/ethnicity(Asian, Indigenous/Alaska Native, Mixed Race, Black/African American, White, and Other/), maternal education (some high school education or less, high school graduate, some college education, college graduate or more), marital status (married, other), the Kotelchuck index of prenatal care adequacy (inadequate, intermediate, adequate, adequate plus).

### Data procedure

The outcome variable for maternal gum disease was recoded into Yes and No. Following the questionnaire instructions, if a question is not applicable, the participant can skip it; “DK/BLANK” or “NA” were recoded into “NO.” Also, since “NA” is not applicable, it was combined with “No” to create a single No value category to represent women who did not self-report gum disease during pregnancy.

Pregnancy outcomes include a small gestational age, categorized as either yes or no, which falls below the 10th percentile. A baby weighing below 2500g (Low Birth Weight) was categorized into three groups: those weighing below 1249g, 1250-1999g, and 2000-2500g. These groups were created by recoding into binary variables, where “YES” became “1” and “NO” became “0”.

Other exposure variables related to maternal oral health, dental visits during pregnancy, Marital Status, and maternal smoking behavior were created by recoding into binary variables, where “yes” became “1” and “no” became “0”.

**Missing variables**. The missing variables include small for gestational age under the 10th percentile (sga_10), maternal race (mat_race), maternal age (mat_age_naphsis), marital status (married), maternal education (mat_ed), prenatal care index (Kotelchuck), Income (pay), and dental cleaning (dds_cln). The “UNKNOWN,” “DK/BLANK,” or “NA” values were excluded, as they comprise less than 5% of the data, to ensure data quality and avoid potential errors in analysis. Subjects missing one or more covariates were dropped from the analysis.

### Data analysis

Data were analyzed using R software version 4.2.1 for analysis [35]. Descriptive statistics were used to describe the demographic characteristics of both the case and control groups. The data were entered, sorted, edited, and cleaned for missing values.

A χ 2 test and frequency analysis were conducted to describe the pregnant women who self-reported gum disease and the control subjects. To assess the relationship between the dependent and independent variables, logistic regression was performed. A *p-value* < 0.01 was observed during bivariate analysis.

## Results

Out of every 100,000 pregnant women, 207 have been identified as having gum disease. This means that gum disease affects 0.207% of the pregnant women in the study population.

### Demographic characteristics

The largest group of pregnant women who self-reported gum disease falls within the 20–24-year age range (32%), compared to 17% in the same age group without gum disease. However, this difference is not statistically significant (p = 0.22). In some cases, the maternal racial distribution indicates a higher percentage of Black/African Americans (27%) and White (Caucasians). In comparison, a lower percentage of Asians (2.5%) were observed in cases compared to those not reporting gum disease (17.7% and 7.7%, respectively) (Table 1). At the same time, no Indigenous/Alaska Natives had reported gum disease

**Table 1:** Demographic Differences Between the Gum Disease Women and the Control Subject.

Characteristics	With gum disease Case group. (n = 162)	Without gum disease (n = 70516)	Without gum disease Control group (n = 468)	P-value
**Age**				
<=17	1(0.6%)	807(1%)	2(0.4%)	0.22
18-19	6(3%)	2199 (3%)	16(3%)	
20-24	**52 (32%)**	12226 (17%)	106(22%)	
25-29	46 (28%)	19454 (28%)	144(29.6%)	
30-34	**35 (22%)**	21680 (31%)	137(28%)	
35-39	17 (10%)	11580 (16%)	65(13.3)	
40+	5 (3%)	2570(3%)	16(3%)	
**Dental Visit**				
Yes	42 (25%)	29614 (42%	160 (32.9%)	0.12
No	**120(74%)**	40902(58%)	326(67.1%)	
**Teeth Cleaned**				
Yes	53 (33%)	29945(42%)	164(33.7%)	0.89
No	**109 (67%)**	40571(58%)	322(66.3%)	
**Prenatal Care Index**				
Adequate	53(32.7%)	31417(44.6%)	198(42.3%)	0.02
Adequate Plus	50(30.9%)	22267(31.6%)	151(32.3%)	
Intermediate	17(10.5%)	9113(12.9%)	63(13.4%)	
Inadequate	**42(25.9%)**	7719(10.9%)	74(15.8%)	
**Marital Status**				
Married	91 (56%)	41792(59%)	232(47.7%)	0.44
Others	71 (44%)	28724(41%)	254(52.3%)	
**Education**				
< 12th grade, no diploma	**33(20%)**	7728(11%)	76(16%)	0.32
Some colleges, no degree/associate’s degree	**51 (31%)**	19477 (28%)	184(37.9%)	
High school grad/GED	62 (38%)	17191(24%)	181(37.2%)	
Bachelor’s/master’s/doctorate/ Professor	15 (9%)	26120(37%)	45(9.3%)	
**Method of Payment**				
Government-funded	**99 (61.1%)**	30482 (43.2%)	287(59.1%)	0.29
Private Insurance	58(35.8%)	35801 (50.8%)	171(35.2%)	
Self-pay	5(3.1%)	4233(6%)	28(5.8%)	
**Maternal Race**				
Asian	4(2.5%)	5434(7.7%)	95 (19.5%)	<0.001
Indigenous/Alaska Native	0	3231 (4%)	260(53.5%)	
Mixed Race	5(7%)	4893 (6%)	44(9.1%)	
Black/African American	**44 (27%)**	12472 (17.7%)	24(4.9%)	
White	97(60.4%)	**39480 (56%)**	27(5.8%)	
Other	1(0.6%)	5006 (7%)	36(7.4%)	
**Smoking**				
Yes	44 (27.2%)	4727 (7%)	132(27.2%)	1
No	**118 (72.8%)**	65789 (93%)	354 (72.8%)	

Values in bold indicate disparities in value.

The majority of women in both groups were married, with a slightly higher percentage among those not reporting gum disease (59%) than those reporting gum disease (56%). A higher percentage of cases had lower levels of education (19% with 9–12 grade, no diploma) compared to those not reporting gum disease (8%). Conversely, a smaller percentage of cases had higher education levels (bachelor/master/doctorate/professional degrees) compared to controls (9% vs. 37%, respectively), as presented in Table 1.

In addition, a smaller percentage of cases reported having a dental visit (25%) or having their teeth cleaned (33%) compared to those not reporting gum disease (42% for both dental visits and teeth cleaning). Similarly, a lower percentage of cases received ‘Adequate’ or ‘Adequate Plus’ prenatal care compared to those not reporting gum disease (32.7% and 30.9% vs. 44.6% and 31.6%, respectively). Therefore, a higher percentage of cases received ‘Inadequate’ prenatal care (25.9%) compared to those not reporting gum disease (10.9%). Furthermore, more cases relied on government-funded healthcare (61.1%) compared to those not reporting gum disease (43.2%). Conversely, a lower percentage of cases (35.8%) had private insurance compared to the controls (50.8%) (Table 1).

### Maternal gum disease and pregnancy outcomes

A higher proportion of Small for Gestational Age (SGA) infants, defined as those below the 10th percentile, was observed among cases with gum disease compared to matched controls (26.5% cases and 14% matched controls), with significant differences between the groups (p < 0.001). However, there was an association between maternal oral gum disease and a baby weighing less than 2500g (p < 0.001) (Table 2).

**Table 2:** Maternal gum disease and pregnancy outcomes.

Variable	Gum disease (%) (n = 162)	Without gum disease (n = 70516)	Without gum disease (%) (n = 486)	P-Value
**Small For Gestational Age Below The 10** ^**th**^ ** Percentile**				
Yes	43(26.5%)	9742 (14%)	63(13%)	<0.001
No	119(73.4%)	60774 (86%)	423(87%)	
Below				
Yes	89(55%)	0	0	<0.001
No	73(45%)	70516(100%)	486(100%)	

The green highlighted column was compared.

A χ 2 test at *p-value* < 0.01.

Mother who had gum disease during pregnancy are 2.42 times more likely to have babies with small for gestational age (SGA) and fall below the 10th percentile (OR = 2.43, 95% CI: 1.56–3.75, p < 0.001), having babies with birthweight below 2500g are 3.51 times higher (OR = 3.51, 95% CI: 2.39–5.16, p < 0.001) compared to those who do not have gum disease (Table 3).

**Table 3:** Maternal gum disease during pregnancy and SGA below the 10th percentile.

Predictors	Coef	SE Coef	Z	p	OR	95% CI
**Small For Gestational Age Below The 10**^**th**^ **Percentile**	0.89	0.22	3.97	0.001	2.43	1.56- 3.75
**Babyweight below 2500g**	1.26	0.120	6.42	0.001	3.51	2.39-5.16

## Discussion

Using the PRAMS dataset, the study provides valuable insights into the multifaceted nature of variation in gum disease among pregnant women. Our study highlights the prevalence of gum disease among pregnant women. Pregnant women between the ages of 20 and 24 years, Black Americans, and those who lack dental visits are prone to gum disease during pregnancy. Additionally, a higher percentage of small for gestational age < 10th percentile was observed in women reporting gum disease during pregnancy, with no significant differences in the prevalence of low birth weight between groups.

Firstly, the prevalence rate of 207 cases per 100,000 indicates that maternal gum disease during pregnancy is a relatively prevalent condition. The low prevalence of self-reported gum disease (0.207%) significantly differs from other studies reporting rates between 24 and42% in pregnant populations [36,37].This discrepancy primarily stems from our reliance on self-reported gum disease status, which inherently underestimates the severity of periodontal conditions. Self-reported assessments tend to underestimate the actual prevalence of periodontal conditions compared to clinical examinations, which typically yield higher prevalence rates [38,39]. The findings highlight the significant prevalence of maternal gum disease during pregnancy, consistent with global trends, and the need for increased awareness and preventive measures.

Although previous studies have consistently reported the highest prevalence of gum disease among pregnant women aged 25–37 years [36,40,41], our study reveals a significant disparity in the prevalence of gum disease among younger pregnant women, particularly in the 20–24- age group. While no significant differences between groups were found for age, a notable difference in the prevalence of reported gum disease was found in the 20–24 years age group. Specifically, 41% of women under 24 years in the gum disease group had gum disease, compared to 21% without gum disease. Additionally, 21% of women in the case group showed less than a high school education (9–12 grade, no diploma) compared to 10% in the control group. These findings highlight the need to further explore the factors influencing gum disease prevalence across different age and education groups, especially given the deviation from the patterns observed in older pregnant women.

Furthermore, our study reveals racial disparities in the prevalence of gum disease among pregnant women. These findings are consistent with other studies that have reported racial disparities in the prevalence of periodontal disease [42–44]. Factors that may contribute to the racial disparities include genetic predisposition, socioeconomic status, access to dental care, and health behaviors like smoking. This might have significant implications for maternal and child health. Therefore, there is a need to address potential disparities among different racial and ethnic groups.

Fewer women reporting gum disease also reported a lower frequency of dental visits and teeth cleaning than those not reporting gum disease. This finding aligns with the broader research landscape, where multiple studies emphasize the importance of regular dental visits and cleanings, particularly for individuals with periodontal disease or a higher risk of developing it [45–47]^.^ The disparity in dental visits and teeth cleanings between those who self-report gum disease highlights the importance of preventive oral health measures in managing gum disease and overall oral health.

Our study findings indicate a significant association between maternal gum disease and an increased risk of having a baby classified as Small for Gestational Age (SGA) below the 10th percentile, and a lower birth weight of less than 2,500 g. The findings from this study are consistent with other observational studies that have reported a link between maternal periodontal disease and adverse pregnancy outcomes like preterm birth, preeclampsia, and fetal growth restriction [29,30]. This finding suggests that maternal gum disease may be an essential risk factor for fetal growth restriction and poor intrauterine growth. Hence, prompt diagnosis and treatment of gum disease during pregnancy may help mitigate these risks.

## Implications of findings

Healthcare providers should consider targeted screening for gum disease, especially among pregnant women and those from racial groups with higher prevalence rates. Tailored counseling and education on oral health and prenatal care are needed, particularly for women with lower education levels and those with gum disease.

Additionally, strategies to enhance dental visits, teeth cleaning, and access to adequate prenatal care are crucial, particularly for women with gum disease. Similarly, collaboration between dental and prenatal healthcare providers is essential in integrating oral health assessments into routine prenatal care. Policymakers should consider expanding access to dental care, particularly for disadvantaged pregnant women, and improving reimbursement for dental services under government-funded healthcare programs.

## Strengths and limitations

The major strength of this study was the use of recent nationally representative PRAMS data. The ability to control for confounding variables enhances the reliability of the findings and strengthens the validity of the study outcomes.

However, some limitations were also observed. Our reliance on self-reported periodontal disease status is a primary limitation, which presents significant methodological challenges. Periodontal diseases are complex infectious and inflammatory conditions that often progress asymptomatically, making patient self-reporting inherently problematic. While patients may notice gingival bleeding, this symptom can be misinterpreted as trauma from routine oral hygiene practices rather than recognized as a sign of disease. Given the “silent” nature of periodontal disease progression, our self-reported prevalence rates likely underestimate the true disease burden in our study population. This measurement limitation affects our ability to quantify the strength of associations between periodontal disease and adverse pregnancy outcomes precisely. While the PRAMS dataset is large and nationally representative, the sample size for gum disease was relatively small. This can limit the statistical power to detect associations between potential determinants and gum disease. Self-reported data introduces potential biases, including recall bias and social desirability bias, which may affect the accuracy of reported findings. The data from both before and after the COVID-19 pandemic are mixed, which may make it difficult to to address the impact of COVID-19. Potential misclassification of exposure and outcome variables may further impact the accuracy and reliability of the study results. Further research and longitudinal studies would be necessary to establish a more definitive understanding of the relationship between pregnancy outcomes and gum disease in this context.

## Conclusion

This study investigated the prevalence of gum disease among pregnant women in the USA, its association with socioeconomic factors, and its relationship with adverse pregnancy outcomes. Our findings revealed a prevalence rate of 207 cases per 100,000 pregnant women nationwide. Regarding socioeconomic factors, we observed significant disparities in prevalence rates, with higher rates among younger women aged 20–24 years, Black women, and those with lower educational attainment (9–12 grade, no diploma). These findings suggest that socioeconomic status plays an essential role in the development of gum disease during pregnancy.

When examining the relationship between gum disease and adverse pregnancy outcomes, we found a significant association between maternal gum disease and small for gestational age (SGA) births and low birth weight (p < 0.001). This suggests that pregnant women with gum disease have a higher risk of delivering babies who are small for their gestational age.

This study highlights the need for targeted maternal oral health interventions, particularly for socioeconomically disadvantaged groups, to mitigate the risk of adverse pregnancy outcomes associated with periodontal disease. The association between gum disease and SGA births highlights the importance of comprehensive prenatal care, including oral health assessments and treatment. Further research is needed to explore the complex relationship between maternal oral health and adverse pregnancy outcomes.
